# Immunohistochemistry (IHC) Versus Genomic Profiling in Cancer: Roles in Precision Medicine

**DOI:** 10.7759/cureus.112007

**Published:** 2026-07-03

**Authors:** Yassir M Elshami, Abuobaida Abukhelaif, Omer A Elrheima, Aamir G Ishaq, Nassir A Ali

**Affiliations:** 1 Histopathology, Laboratory and Blood Bank, King Khaled Hospital, Najran, SAU; 2 Medicine, Al Baha University, Al-Baha, SAU; 3 Pathology, Jazan University, Jazan, SAU; 4 Histopathology, Regional Laboratory &amp; Central Blood Bank, Jazan Health Cluster, Jazan, SAU; 5 Medical Laboratory Technology, Directorate of Laboratories and Blood Banks, Wad Medani, SDN

**Keywords:** actionable mutations, biomarker discordance, genomic profiling, immunohistochemistry, next-generation sequencing, precision medicine, precision oncology

## Abstract

Precision oncology increasingly depends on accurate biomarker assessment to guide targeted therapies. Immunohistochemistry (IHC) has long been central to routine pathology due to its accessibility and diagnostic utility; however, the expanding role of molecularly driven treatments has highlighted limitations in protein-based testing. Genomic profiling, using next-generation sequencing (NGS), enables direct detection of genetic alterations that may not be reflected at the protein level. This review explored how IHC and molecular diagnostic approaches contribute to tumour diagnostic accuracy and therapeutic management across solid tumours. The review process framework followed the Preferred Reporting Items for Systematic reviews and Meta-Analyses (PRISMA) 2020 statement. Literature retrieved from PubMed, Scopus, and Web of Science yielded 15 published studies comparing IHC and genomic profiling methods in precision oncology. Genomic profiling consistently identified clinically actionable alterations that were missed or misclassified by IHC, including gene fusions, exon-skipping events, copy-number alterations, and pathogenic mutations lacking reliable protein-level surrogates. High discordance was observed for anaplastic lymphoma kinase (ALK), proto-oncogene 1, receptor tyrosine kinase (ROS1), mesenchymal-epithelial transition gene exon 14 skipping alteration (MET exon 14), human epidermal growth factor receptor 2 (HER2), mismatch repair status, and phosphatase and tensin homolog (PTEN). Across multiple tumour types, genomic testing refined molecular classification and expanded eligibility for targeted and immunotherapies beyond IHC-based assessment. The evidence reviewed indicates that genomic technologies expand tumour characterisation beyond conventional protein-based biomarkers by detecting mutations, copy-number changes, gene fusions, and other actionable molecular events. While IHC remains valuable for initial screening, integration of genomic testing is essential for accurate biomarker assessment and optimal treatment selection in modern precision oncology.

## Introduction and background

Continuous progress in pathology and molecular testing during the last two decades has transformed the diagnostic and therapeutic landscape of modern oncology. Despite these achievements, important challenges persist, highlighting the need for ongoing innovation in cancer diagnostics [1]. Although traditionally, cancer diagnosis depends mainly on histomorphology and immunohistochemistry (IHC) for tumour classification and biomarker assessment, increasing recognition of the molecular diversity of cancers has revealed limitations of morphology-based assessment alone, prompting the integration of genomic profiling to improve diagnostic precision and guide targeted therapies [2]. In parallel, digital pathology and artificial intelligence-assisted image analysis have emerged as important adjuncts in oncological diagnostics, facilitating improved tumour assessment and contributing to the broader implementation of precision oncology strategies [3]. These tools are essential, as accurate diagnosis depends on identifying tumour characteristics, immune markers, patterns of differentiation, and specific lineage signatures is crucial for making accurate diagnoses. IHC has held a crucial position in detecting important biomarkers, including hormone receptors in breast cancer, mismatch-repair proteins (MMR) in colorectal cancer, and programmed cell death ligand 1 (PD-L1) expression for choosing immunotherapy [4-6]. Owing to its broad accessibility, fast processing time, and cost-effectiveness, IHC remains a fundamental tool in diagnostic procedures worldwide, including regions with limited laboratory infrastructure [7]. Nevertheless, the emergence of precision medicine has reshaped contemporary approaches to cancer classification, prognostication, and treatment selection. The biological behaviour of tumours, how well treatments work, and patient survival rates are increasingly influenced by the molecular changes that drive tumour development [1,8]. Genomic profiling, enhanced by next-generation sequencing (NGS), has established a new framework whereby mutations, variations in gene copies, gene fusions, and distinct mutational patterns offer essential therapeutic insights that cannot be obtained solely from histology or IHC [9,10].

The emergence and rapid evolution of biomarker-directed therapies, including epidermal growth factor receptor (EGFR)-, anaplastic lymphoma kinase (ALK)-, proto-oncogene 1, receptor tyrosine kinase (ROS1)-directed therapies in lung cancer, B-Raf proto-oncogene, serine/threonine kinase (BRAF)-targeted agents in melanoma and colorectal carcinoma, DNA repair-targeted strategies for breast cancer susceptibility gene (BRCA)-altered tumours, and tumour-agnostic agents such as neurotrophic tyrosine receptor kinase (NTRK) inhibitors, have substantially increased the clinical importance of genomic biomarker testing for therapeutic decision-making [11]. These clinical advancements have transformed cancer management from a predominantly organ-based approach towards molecularly informed precision oncology, in which genomic profiling complements conventional pathology for tumour classification, prognostication, and treatment selection [11,12]. For many cancers, accurate diagnosis and optimal therapeutic decision-making require integration of genomic alterations with protein expression and histomorphological findings [4,11]. Consequently, understanding the complementary roles of IHC and genomic profiling is fundamental to an integrated diagnostic framework in modern precision oncology [11]. In breast cancer, for instance, the difference between human epidermal growth factor receptor 2 (HER2) expression and Erb-B2 receptor tyrosine kinase 2 (ERBB2) amplification carries important treatment consequences [13]. In a similar manner, PD-L1 IHC indicates protein levels, while tumour mutational burden (TMB) and certain genetic markers affect the reaction to immune checkpoint inhibitors [14,15].

Current clinical guidelines recommend tumour-specific algorithms for biomarker assessment that integrate IHC with complementary molecular techniques when appropriate. For HER2 testing in breast cancer, IHC is performed as the initial assay, with equivocal (2+) cases undergoing confirmatory in situ hybridisation (ISH/FISH) to determine ERBB2 gene amplification according to American Society of Clinical Oncology (ASCO)/College of American Pathologists recommendations. In gastric and gastro-oesophageal adenocarcinomas, HER2 interpretation also relies on tumour-specific IHC scoring criteria with confirmatory ISH for equivocal cases because of greater intratumoral heterogeneity. For PD-L1, IHC remains the standard predictive assay; however, the scoring system varies according to tumour type, including the Tumour Proportion Score (TPS) in non-small cell lung cancer and the Combined Positive Score (CPS) in gastric, oesophageal, and several other malignancies, with treatment eligibility determined by tumour-specific guideline thresholds [6,14,15]. These guideline-based algorithms illustrate how IHC serves as the foundation of biomarker assessment, whereas complementary molecular testing is incorporated when additional diagnostic or predictive information is required.

Although it is becoming more advanced, genomic profiling presents difficulties such as high costs, restricted accessibility, inconsistent bioinformatics assistance, and extended turnaround periods [16]. Various cancer types demonstrate the added benefit of utilising both methods. In colorectal cancer, the use of MMR protein IHC is common for screening Lynch syndrome. However, genomic testing offers a conclusive classification of microsatellite instability (MSI) and identifies BRAF V600E mutations, which are important for prognosis and treatment strategies [17]. Lung cancer illustrates the crucial importance of genomic profiling in detecting various actionable driver mutations, while IHC aids in evaluating PD-L1 and classifying tumours [18]. In lymphomas and other blood cancers, IHC helps identify the type of cells and their development, while genetic abnormalities assist in determining risk levels and directing specific treatments [19]. Considering these complexities, a combined evaluation of both IHC and genomic profiling is increasingly regarded as the standard approach. Nevertheless, healthcare professionals and pathologists frequently encounter uncertainty regarding the indications in which genomic profiling ought to be conducted, its additional benefits compared to IHC, and its actual effects on patient outcomes. Moreover, healthcare systems with differing levels of resources may find it challenging to provide complete genomic testing for every patient, requiring prioritisation based on evidence [12,16-19].

This review examines the complementary roles of IHC and genomic profiling in the diagnosis and management of major cancers. By evaluating their diagnostic performance, therapeutic relevance, areas of concordance and discordance, and clinical impact, the review highlights how these approaches can be integrated to support precision oncology and improve patient care. The results also emphasise important factors for implementing healthcare in low- and middle-income countries, where access to advanced molecular technologies is growing but still not available to everyone.

## Review

Material and methods

Study Design

The review was structured using the Preferred Reporting Items for Systematic reviews and Meta-Analyses (PRISMA) 2020 reporting framework recommendations [20] and methodological principles outlined in the Cochrane Handbook for Systematic Reviews of Interventions [21] to evaluate the complementary roles of IHC and genomic profiling in cancer diagnosis and precision oncology. Emphasis was placed on diagnostic accuracy, therapeutic implications, concordance and discordance patterns, and their impact on clinical decision-making. As the review was initiated without a prospectively developed protocol, it was not registered in PROSPERO or any other systematic review registry. Consequently, no protocol modifications or amendments were required during the conduct of the review. The review involved systematic literature searching, study screening, data extraction, Critical Appraisal Skills Programme (CASP)-based quality assessment [22], and qualitative synthesis of 15 eligible comparative studies.

Data Sources and Search Strategy

Searched databases: An extensive search was undertaken to identify studies evaluating IHC with genomic profiling approaches in cancer diagnostics and precision oncology. The search was done across PubMed, Scopus, and Web of Science to ensure broad coverage of the available evidence. Search strategies incorporated both controlled vocabulary and free-text terms related to IHC biomarkers, molecular pathology, genomic testing, NGS technologies, and precision oncology. Search terms were combined using Boolean operators (AND, OR) to enhance outputs and improve the relevance of identified studies.

Search keywords: Key search terms included: “immunohistochemistry AND genomic profiling”, “IHC AND next-generation sequencing”, “IHC AND NGS AND concordance”, “HER2 AND sequencing AND discordance”, “ALK rearrangement AND NGS”, “mismatch repair AND IHC AND MSI sequencing”, and “precision oncology AND genomic profiling”.

Eligibility timeframe: Eligibility was limited to English-language studies involving human participants that had undergone peer review. The final database search was performed on 31 December 2025. Studies published between January 2013 and December 2025 were considered eligible for inclusion. The resulting report collection was used to support duplicate detection, record screening, eligibility determination, and study selection procedures across all review stages.

Study Selection and Eligibility Criteria

All retrieved records were imported into a reference management system, and duplicate entries were removed. All identified records were first evaluated through title and abstract assessment before full-text review. Studies considered potentially relevant were subsequently reviewed in full-text evaluation according to the predefined criteria. Study selection was performed manually by the review authors, and no automated screening or eligibility assessment tools were used.

Inclusion criteria: Only studies that reported a direct comparison between IHC and at least one genomic approach, including NGS, comprehensive genomic profiling, RNA sequencing, or sequencing-based MSI assessment, were included in the review.

Exclusion criteria: Non-comparative studies, conference abstracts, narrative reviews, methodological papers lacking clinical comparison, and studies evaluating only a single diagnostic modality (IHC or genomic testing alone) were excluded. Full-text exclusions were recorded with reasons according to the predefined eligibility criteria.

Study Selection Process

**Figure 1:**
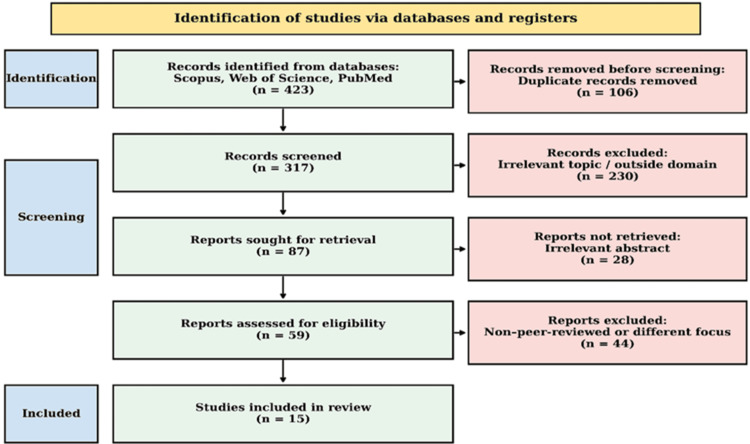
PRISMA 2020 flow diagram PRISMA: Preferred Reporting Items for Systematic reviews and Meta-Analyses; The diagram outlines the study identification, screening, eligibility assessment, and final inclusion of 15 studies in the qualitative synthesis in accordance with PRISMA 2020 recommendations [19].

PRISMA 2020 flow diagram: The process used to identify, screen, and select eligible studies was conducted in accordance with the PRISMA 2020 reporting framework and is illustrated in Figure 1.

Database searches yielded 423 records. After removing 106 duplicates, 317 citations underwent initial screening, resulting in the exclusion of 230 records. Eighty-seven articles were considered potentially relevant and reviewed for retrieval, of which 28 were unavailable or unsuitable for eligibility assessment. The remaining 59 full-text articles were evaluated, and 44 were excluded after detailed review. A list of excluded full-text studies and the corresponding reason for exclusion is provided in the appendix. Ultimately, 15 studies satisfied the eligibility criteria and were included in the qualitative synthesis.

Immunohistochemistry (IHC) Assessment

IHC was evaluated as the index or reference diagnostic method across included studies. Extracted data included antibody clones, staining platforms, and scoring systems where reported.

Evaluated biomarkers: Commonly assessed biomarkers included ALK, HER2, estrogen/progesterone receptor (ER/PR), Ki-67, MMR proteins (MLH1, MSH2, MSH6, PMS2), p53, phosphatase and tensin homolog (PTEN), and PD-L1, reflecting established diagnostic practice across tumour types [4-7,13,17,18].

IHC scoring systems: Studies employing clinically validated antibodies and tumour-specific scoring algorithms were prioritised, reflecting routine diagnostic laboratory practice [5,6,13].

Genomic Profiling and Molecular Testing

To characterise tumour molecular profiles, the included studies employed a variety of genomic technologies, including targeted DNA NGS panels, RNA sequencing, gene expression profiling, copy-number variation analysis, and sequencing-based MSI assessment. Data extraction focused on key technical and analytical parameters, such as sequencing platforms, panel composition, analytical scope, and the types of genomic alterations detected. Attention was given to the ability of these methodologies to uncover therapeutically actionable molecular abnormalities - including mutations, gene fusions, exon-skipping events, copy-number changes, and MSI status [9-12,19] - that may be overlooked by protein-based assays. This capability was especially relevant in biologically heterogeneous tumours, where IHC findings do not always accurately reflect the underlying genomic landscape.

Data Extraction

Data extraction was performed independently by two reviewers using a predefined extraction framework. Any discrepancies were resolved through discussion and consensus. No automation tools were used during the data extraction process. Information from eligible studies was collected and organized by the review team using a predefined extraction framework designed to ensure consistency and accuracy across the included studies. Extracted variables from each study comprised tumour type, sample size, biomarker(s) assessed, details of the IHC methodology (including antibody selection and scoring criteria where available), genomic profiling technique employed (such as NGS platform, panel composition, or sequencing strategy), diagnostic performance measures, concordance or discordance between immunohistochemical and genomic findings, and the reported clinical or therapeutic significance of the results. The extracted information was systematically reviewed to identify clinically relevant differences between IHC and genomic profiling. Subsequently, the data were synthesised into structured summary tables outlining study characteristics, diagnostic agreement and disagreement patterns, methodological strengths and limitations, and the overall clinical impact reported by the included studies [20,21].

Quality Assessment

Methodological quality and risk of bias were assessed independently by two reviewers using the CASP Diagnostic Test Accuracy Checklist [22]. Disagreements were resolved through discussion and consensus. Owing to substantial methodological heterogeneity among the included studies, was applied as a qualitative appraisal framework rather than a numerical scoring system. No automation tools, machine-learning methods, or artificial intelligence-assisted software were used during the assessment process. Publication bias was not formally evaluated because no meta-analysis was performed. Grading of Recommendations Assessment, Development and Evaluation (GRADE) assessment was not performed due to substantial study heterogeneity; methodological quality was assessed qualitatively using the CASP checklist [22].

Data Synthesis and Statistical Analysis

Because the included studies differed considerably in their objectives, patient cohorts, molecular testing strategies, and reported outcomes, formal statistical synthesis was not feasible. The results were therefore examined qualitatively to explore diagnostic performance, concordance patterns, sources of discordance, and their relevance to precision oncology practice [23].

Reproducibility Statement

Study selection was undertaken through a structured multi-stage review process involving record identification, title and abstract screening, full-text evaluation, and final study inclusion, using PRISMA 2020 reporting standards to ensure transparency and reproducibility throughout the review.

Results

A total of 15 comparative studies evaluating immunohistochemistry and genomic profiling across multiple solid tumour types were included in the qualitative synthesis following PRISMA-guided study selection. The results are presented under study characteristics, quality assessment, IHC findings, genomic profiling findings, and concordance and discordance patterns.

Study Characteristics

Following the selection process, 15 eligible comparative studies evaluating immunohistochemistry and genomic profiling were retained for qualitative analysis. The principal characteristics of these investigations are shown in Table 1.

**Table 1:** Characteristics of the included studies comparing immunohistochemistry and genomic profiling (n=15) This table presents the characteristics of the 15 studies included in the review, including tumour types, biomarkers evaluated, immunohistochemical and genomic testing methods, and the reported diagnostic agreement between these approaches. The studies covered multiple solid tumours, including lungs, breast, gastric, colorectal, and endometrial cancers, as well as carcinoma of unknown primary and pan-cancer cohorts. Overall, the findings illustrate the complementary contributions of immunohistochemistry and genomic profiling to precision oncology. IHC, immunohistochemistry; NGS, next-generation sequencing; RNA-seq, RNA sequencing; CNV, copy-number variation; MSI, microsatellite instability; MSS, microsatellite stable; MMR, mismatch repair; ER, estrogen receptor; PR, progesterone receptor; HRs, hormone receptors; CD, Cluster of differentiation; CUP, carcinoma of unknown primary; CDX2: Caudal Type Homeobox 2; GE, gastroesophageal; PPV, positive predictive value; ISH, in situ hybridization; RT-qPCR, reverse transcription quantitative polymerase chain reaction; AUC, area under the receiver operating characteristic curve; ICC, intraclass correlation coefficient; κ, Cohen's kappa; PD-L1, programmed cell death ligand 1; TMB, tumour mutational burden; ALK, Anaplastic Lymphoma Kinase; ROS1, ROS Proto-Oncogene 1, Receptor Tyrosine Kinase; MET exon 14, Mesenchymal-Epithelial Transition (MET) Gene Exon 14 Skipping Alteration; HER2, Human Epidermal Growth Factor Receptor 2; PTEN, Phosphatase and Tensin Homolog; EGFR, Epidermal Growth Factor Receptor; BRAF, B-Raf Proto-Oncogene, Serine/Threonine Kinase; BRCA, Breast Cancer Susceptibility Gene;  NTRK,  Neurotrophic Tyrosine Receptor Kinase; ERBB2, Erb-B2 Receptor Tyrosine Kinase 2; NRAS, Neuroblastoma RAS viral oncogene; CRC, Colorectal Cancer; NSCLC, Non-Small Cell Lung Cancer; PCR, polymerase chain reaction. Sample sizes are as reported in the original studies.

No.	Author (Year)	Sample size	Cancer type	Biomarker(s)	IHC method	Genomic method	Concordance/discordance summary
1	Nong et al. (2019) [24]	107	NSCLC	EGFR, ALK, ROS1	EGFR hotspot IHC; ALK/ROS1 IHC	Targeted NGS	Overall concordance percentage was not reported. NGS provided more comprehensive and reliable detection than IHC, especially for EGFR exon 19 alterations. NGS increased ALK rearrangement detection and reduced false-positive ROS1 IHC results.
2	Zeng et al. (2020) [25]	472 NSCLC pt; 319 received crizotinib; 76 do both assays	NSCLC	ALK	Ventana ALK (D5F3)	NGS fusion panel	Overall concordance: 78.9%. Discordant cases included 18.4% NGS+/IHC− and 2.6% IHC+/NGS−.
3	Niu et al. (2020) [26]	Breast n=280; gastric n=50	Breast & gastric	HER2	HER2 IHC ± FISH	NGS CNV	Breast cancer: concordance 97.3% (sensitivity 95.4%; specificity 98.7%). Gastric cancer: moderate concordance (sensitivity 64.7%).
4	Kim et al. (2023) [27]	18	Endometrial, brain, ovarian, lung, unknown primary cancers	PTEN	PTEN IHC	NGS panel	Overall concordance was not reported. PTEN missense mutations showed variable immunostaining. Complete PTEN loss occurred in 5/18 tumours, while PTEN R130 mutations did not consistently correlate with IHC findings. The study supports complementary use of IHC and NGS for accurate PTEN status assessment.
5	Xiao et al. (2021) [28]	430; triple-tested subset n=98	Colorectal	MSI/MMR, TMB, KRAS, NRAS, BRAF, HER2	MLH1, PMS2, MSH2, MSH6	381-gene targeted NGS panel for MSI and TMB analysis; PCR-based MSI testing for validation	NGS-MSI showed very high concordance with PCR (99.0%) and high concordance with IHC (93.9%), with 100% specificity against both methods. NGS resolved four of seven IHC-PCR discordant cases and identified additional MSS-TMB-high tumours, supporting its complementary diagnostic and predictive value.
6	Sorokin et al. (2020) [29]	Breast n=39; Lung n=19	Breast & lung	HER2/ERBB2, ER/ESR1, PR/PGR, PD-L1/CD274	Standard IHC	RNA-seq	Overall concordance was not reported. RNA sequencing showed strong correlation with IHC for HER2, ER, PR, and PD-L1 (AUC 0.912–0.963), supporting RNA-seq as a complementary method for biomarker assessment from FFPE tissues.
7	Kıvrak et al. (2022) [30]	256 NSCLC screened; 26 selected for detailed analysis	NSCLC	HER2	HER2 IHC	NGS CNV	Overall concordance was not reported. HER2 IHC positivity was higher in NGS-amplified than non-amplified cases (50% vs 23%). NGS showed strong agreement with SISH (ICC r = 0.777–0.805), although three NGS-non-amplified cases were SISH amplified, supporting complementary confirmatory testing.
8	Rios-Doria et al. (2023) [31]	2115	Endometrial	POLE mutation, TP53 mutation, MSI/MMR status, p53	p53/MMR IHC	NGS with POLE mutation analysis, TP53 mutation analysis, and MSI sensor scoring	Overall concordance was high (κ = 0.962). Integrated NGS and IHC classified 87% of endometrial carcinomas versus 66% with the IHC-based surrogate alone. Discordant cases were primarily TP53-mutated tumours with normal p53 IHC, supporting complementary genomic profiling.
9	Shah et al. (2022) [32]	176	Breast cancer, NSCLC, CRC, GE ca, Pancreatic, Uterine ca	ERBB2	HER2 IHC ± FISH	Tissue & ctDNA NGS	ERBB2 copy-number gain by NGS showed high PPV for HER2 positivity, particularly in breast cancer. PPV was 88% for tissue NGS and 80% for ctDNA overall, increasing to 97% and 93%, respectively, in breast cancer. Lower PPV in non-breast cancers reflected discordance between ERBB2 amplification and HER2 protein overexpression.
10	Handorf et al. (2013) [33]	157	Metastatic tumours with an uncertain primary site	Tissue-of-origin gene expression markers	Sequential IHC panel using up to 84 stains	Gene expression profiling (Path work Tissue of Origin Test)	Gene expression profiling showed higher diagnostic accuracy than sequential IHC overall (89.2% vs 83.3%) and in poorly differentiated/undifferentiated carcinomas (91% vs 71%). GEP also outperformed IHC when a second round of stains was required (83.1% vs 67.0%), supporting its complementary role in metastatic tumours of uncertain primary.
11	Choi et al. (2022) [34]	CRC n=225; NSCLC n=109; breast n=260; gastric n=64	CRC, NSCLC, Breast, and Gastric	KRAS, NRAS, BRAF, EGFR, ALK, ROS1, ERBB2	Orthogonal including IHC and ISH/FISH for ALK, ROS1, and ERBB2 assessment	K-MASTER targeted NGS panel	NGS showed high agreement with orthogonal methods, although concordance varied by biomarker. Concordance was 100% for ALK, 95.2% for ROS1, 94.5% for EGFR, and 85.0% (breast) and 93.8% (gastric) for ERBB2. Lower sensitivity for ROS1 and ERBB2 supports complementary orthogonal testing.
12	Kushnarev et al. (2025) [35]	365	Breast, lung, gastrointestinal, and other solid tumours	ER1, PR, AR, MKI67, ERBB2, CD274 (PD-L1), CDX2, KRT7, KRT20	Standard IHC	RNA–seq	Overall concordance percentage was not reported, but RNA-seq demonstrated strong agreement with IHC, showing Spearman correlations of 0.53–0.89, ROC AUC values of 0.84–0.99, and diagnostic accuracy of up to 98% for selected biomarkers. PD-L1 showed only moderate correlation (ρ = 0.63), reflecting tumour microenvironment and purity effects.
13	Sinn et al. (2017) [36]	Not specified in abstract	Breast	ER, PR, Ki–67	IHC (visual & digital)	RT-qPCR	ER and PR showed excellent concordance between RT-qPCR and IHC (91.4–96.6%), whereas Ki-67 demonstrated only moderate correlation (Spearman's r=0.47–0.56). RT-qPCR showed superior specificity for predicting pathological complete response.
14	Ali-Fehmi et al. (2024) [37]	191767	Solid tumours (CRC, GE, small bowel, endo, and other cancers)	MMR/MSI	MMR IHC	NGS–MSI	Very high concordance (99.69%; discordance 0.31%). NGS-MSI identified MSI-H tumours missed by IHC, while IHC detected MMR-deficient tumours missed by NGS, supporting complementary testing.
15	Aydın Meriçöz et al. (2025) [38]	139	Mixed solid tumours including CRC, pancreatic, cholangioca, NSCLC, gastric, ovarian, and others	MMR/MSI	MMR IHC	NGS–MSI	99.0% concordance (κ=0.93); NGS identified two MSI-H tumours missed by MMR IHC, supporting complementary testing.

The reviewed studies in this review represented a highly diverse patient population and collectively encompassed more than 196,000 tumour specimens derived from a wide range of malignancies, including non-small cell lung carcinoma (NSCLC), breast, gastric, colorectal, endometrial, carcinoma of unknown primary (CUP), and pan-cancer cohorts. The substantial variation in tumour types and cohort sizes provided a broad perspective on the comparative performance of immunohistochemistry and genomic profiling across different clinical settings.

Sample sizes varied markedly, ranging from small analytical validation cohorts (n=18) to large population-based datasets exceeding 190,000 cases, underscoring heterogeneity in study scale and statistical power [24-38]. Most studies employed retrospective comparative diagnostic designs, with geographical representation from Asia, Europe, and North America, supporting broad external validity across healthcare systems and laboratory practices [24-38]. The most frequently interrogated biomarkers were HER2/ERBB2, ALK, EGFR, mismatch repair (MMR)/MSI, hormone receptors (ER/PR), p53, PTEN, and PD-L1, reflecting contemporary priorities in precision oncology and predictive biomarker testing [24-32,34-38].

Study Quality and Risk of Bias Assessment

The evaluated studies represented a heterogeneous but informative evidence base. Variations in tumour characteristics, laboratory methodologies, sequencing platforms, and reporting standards were evident across investigations. Several studies were retrospective, introducing an inherent risk of selection bias; however, most employed recognised diagnostic approaches and clearly defined outcome measures. Taken together, the methodological characteristics of the included studies were considered adequate for narrative synthesis and comparative interpretation of findings. Detailed study-level quality assessments and risk of bias evaluations are presented in the appendices. Evidence certainty was not formally assessed; methodological quality was evaluated using the CASP checklist.

Immunohistochemical Findings

IHC served as the cornerstone diagnostic modality across all included studies and was routinely employed either as the primary biomarker assessment tool or as a comparator for genomic testing. The studies utilised established, clinically validated antibodies and tumour-specific scoring systems for biomarkers including ALK (Ventana D5F3), HER2, ER, PR, Ki-67, DNA MMR markers (MLH1, MSH2, MSH6, and PMS2), together with p53, PTEN, and PD-L1, reflecting routine diagnostic practice in surgical pathology laboratories [24,25,27,30].

Across tumour types, IHC demonstrated high sensitivity and clinical reliability for well-established biomarkers, particularly ER, PR, HER2 in breast cancer, as well as MMR proteins in colorectal and endometrial carcinomas. In these settings, protein expression generally correlated well with clinically relevant molecular alterations, supporting the continued role of IHC as a rapid, cost-effective, and widely accessible first-line testing strategy [25,35,36]. However, important limitations were identified across multiple tumour types. Several studies reported inter-observer variability, challenges in interpreting equivocal staining patterns, and reduced predictive accuracy in biologically heterogeneous tumours, particularly gastric carcinoma and non-breast solid tumours [25,31,35]. HER2 and Ki-67 assessment were especially susceptible to scoring variability, while PTEN and PD-L1 expression did not consistently reflect the underlying genomic landscape. These findings highlight a fundamental limitation of protein-based assays: protein expression may be influenced by post-transcriptional regulation, tumour heterogeneity, and technical factors, resulting in discordance between immunophenotypic and genomic findings [26,31]. These observed discrepancies suggest that IHC alone may be insufficient for comprehensive biomarker characterisation in selected clinical scenarios. For example, several studies demonstrated that genomic profiling identified actionable alterations in tumours classified as negative or equivocal by IHC, thereby expanding eligibility for targeted therapies and improving molecular stratification. Similarly, discordant MMR/MSI cases emphasised the value of molecular confirmation. These findings indicate that sequencing-based MSI assessment may serve as a valuable confirmatory tool in cases showing equivocal or discordant MMR IHC, particularly when therapeutic decisions depend on accurate MSI status determination. Collectively, the evidence supports an integrated diagnostic approach in which IHC functions as an efficient screening modality, while genomic profiling provides additional molecular resolution in diagnostically challenging or clinically consequential cases.

Genomic Profiling Findings

The studies included in this review utilised diverse molecular testing platforms, encompassing targeted next-generation DNA sequencing panels, RNA sequencing, gene-expression analyses, copy-number variation assessment, and sequencing-based MSI testing [24,28,29,33,36,37]. These technologies enabled comprehensive molecular assessment of tumours by simultaneously detecting multiple categories of molecular abnormalities, including sequence mutations, gene rearrangements, copy-number alterations, and MSI-related changes, many of which may not be reliably identified through IHC alone. Across several tumour types, genomic profiling provided additional clinically relevant findings that refined molecular classification and facilitated the identification of therapeutically actionable targets [24,25,28,33].

Among the evaluated biomarkers, NGS-based MSI assessment demonstrated particularly strong concordance with conventional MMR IHC, with reported discordance rates as low as 0.31%. Importantly, genomic testing identified a small subset of MSI-high tumours that retained MMR protein expression by IHC, thereby preventing potential false-negative classifications and expanding eligibility for immune checkpoint inhibitor therapy. These findings suggest that sequencing-based MSI assessment may serve as a valuable complementary or confirmatory approach in cases with equivocal, discordant, or clinically unexpected MMR IHC results, particularly when treatment decisions depend on accurate MSI determination [36,37].

RNA-based molecular approaches also enabled reliable and objective assessment of established biomarkers, including hormone receptors and HER2. Unlike conventional semi-quantitative visual scoring, RNA-based assays provide continuous and objective measurements of gene expression, potentially reducing observer variability and improving reproducibility. Collectively, these observations indicate that genomic profiling not only complements traditional IHC evaluation but may also overcome some of its inherent limitations, particularly in biologically heterogeneous tumours and in settings where precise molecular characterization is required for therapeutic decision-making [28,34,35].

Concordance and Discordance Patterns

Overall diagnostic agreement between IHC and genomic profiling varied according to both the biomarker and tumour type. Reported concordance ranged from 78.9% to 99.7%, with the highest agreement observed for MMR/MSI, ALK, and HER2 assessment in breast cancer, whereas lower agreement or variable correlation was reported for PTEN, PD-L1, and HER2 in gastric and other non-breast tumours. These findings are summarised in Table 2.

**Table 2:** Concordance pattern, clinical impact, and diagnostic utility of immunohistochemistry and genomic profiling across included studies (n=15) ALK, anaplastic lymphoma kinase; AUC, area under the receiver operating characteristic curve; CNV, copy-number variation; CRC, colorectal cancer; ctDNA, circulating tumour DNA; CUP, carcinoma of unknown primary; EGFR, epidermal growth factor receptor; ER, estrogen receptor; HER2, human epidermal growth factor receptor 2; ICC, intraclass correlation coefficient; IHC, immunohistochemistry; κ, Cohen's kappa coefficient; MMR, mismatch repair; MSI, microsatellite instability; MSS, microsatellite stable; NGS, next-generation sequencing; NSCLC, non-small cell lung cancer; PCR, polymerase chain reaction; PD-L1, programmed death-ligand 1; PPV, positive predictive value; PR, progesterone receptor; RNA-seq, RNA sequencing; ROS1, ROS proto-oncogene 1 receptor tyrosine kinase; SISH, silver in situ hybridisation; TMB, tumour mutational burden; TP53, tumour protein p53; TPS, tumour proportion score.

No.	Author (Year)	Tumour Type	Biomarker(s)	Diagnostic Agreement (Concordance/Discordance)	Clinical Impact	Advantages	Limitations
1	Nong et al. (2019) [24]	NSCLC	EGFR, ALK, ROS1	Overall concordance not reported. NGS detected additional EGFR, ALK and ROS1 alterations, reducing false-positive ROS1 IHC.	Improved targeted therapy selection	IHC rapid; NGS comprehensive	IHC false positives; NGS cost
2	Zeng et al. (2020) [25]	NSCLC	ALK	78.9% concordance; NGS identified additional ALK-positive cases.	Improved crizotinib candidate identification	IHC accessible; NGS sensitive	IHC may miss rare fusions
3	Niu et al. (2020) [26]	Breast & Gastric	HER2	97.3% concordance in breast cancer; moderate concordance in gastric cancer (sensitivity 64.7%).	Reduced equivocal HER2 classification	Guideline-based IHC; NGS CNV assessment	Gastric heterogeneity
4	Kim et al. (2023) [27]	Mixed tumours	PTEN	Overall concordance not reported; variable PTEN IHC expression with NGS-confirmed mutations.	Avoided PTEN misclassification	IHC accessible; NGS definitive	IHC poor mutation detection
5	Xiao et al. (2021) [28]	CRC	MSI/MMR, TMB	99.0% concordance with PCR and 93.9% with IHC; NGS resolved discordant MSI/MMR cases.	Optimised immunotherapy selection	Low-cost IHC; NGS MSI+TMB	Infrastructure demands
6	Sorokin et al. (2020) [29]	Breast & Lung	HER2, ER, PR, PD-L1	Overall concordance not reported; strong RNA-seq/IHC agreement (AUC 0.912–0.963).	Quantitative biomarker assessment	RNA-seq objective; IHC spatial	RNA-seq standardisation
7	Kıvrak et al. (2022) [30]	NSCLC	HER2	Overall concordance not reported; strong NGS-SISH agreement (ICC r = 0.777–0.805) with partial discordance.	Accurate HER2 amplification detection	ISH/NGS reliable	IHC low predictive value
8	Rios-Doria et al. (2023) [31]	Endometrial	POLE, TP53, MSI/MMR	High concordance (κ = 0.962); integrated testing classified 87% vs 66% with IHC alone.	Refined molecular classification	IHC TCGA surrogate	NGS cost, TAT
9	Shah et al. (2022) [32]	Multiple tumours	ERBB2	High PPV for HER2 positivity (88% tissue NGS; 80% ctDNA), increasing to 97% and 93% in breast cancer.	HER2 stratification	NGS broad screening	Lower PPV outside breast
10	Handorf et al. (2013) [33]	CUP	Tissue-of-origin markers	Higher diagnostic accuracy (89.2% vs 83.3% overall).	Improved CUP diagnosis	High molecular accuracy	IHC labour-intensive
11	Choi et al. (2022) [34]	Multiple tumours	Kras, NRAS, BRAF, EGFR, ALK, ROS1	Concordance varied by biomarker (ALK 100%, ROS1 95.2%, EGFR 94.5%).	Supported precision oncology	NGS multi-gene	IHC single-marker
12	Kushnarev et al. (2025) [35]	Mixed tumours	Multiple biomarkers	Overall concordance not reported; strong agreement (Spearman ρ = 0.53–0.89; accuracy up to 98%).	Objective biomarker thresholds	RNA-seq quantitative	Tumour purity effects
13	Sinn et al. (2017) [36]	Breast	ER, PR, Ki-67	ER/PR concordance 91.4–96.6%; Ki-67 showed moderate correlation.	Better pCR prediction	RT-qPCR sensitivity	Cost, availability
14	Ali-Fehmi et al. (2024) [37]	Pan-cancer	MSI/MMR	99.69% concordance (0.31% discordance).	Identified MSI-H missed by IHC	NGS-MSI sensitivity	Bioinformatics needs
15	Aydın Meriçöz et al. (2025) [38]	Mixed tumours	MSI/MMR	99.0% concordance (κ = 0.93); NGS detected MSI-H tumours missed by IHC.	Improved ICI selection	Combined testing	Cost, infrastructure

High concordance was consistently observed for MMR deficiency and MSI, as well as for hormone receptor assessment in breast cancer, supporting the continued utility of IHC as a reliable first-line screening tool in these settings. Conversely, biomarkers such as ALK, ROS1, HER2 in non-breast malignancies, PTEN, and PD-L1 demonstrated variable concordance, reflecting the complex relationship between genomic alterations and protein expression [24-27,30,32,37].

Several studies identified clinically significant discordant cases in which genomic profiling detected actionable molecular alterations despite negative or inconclusive IHC findings. This was particularly evident in ALK-rearranged NSCLC, HER2-amplified tumours, and MSI-high cancers, where sequencing-based approaches identified patients who may otherwise have been excluded from potentially beneficial targeted or immune-based therapies [24-26,32,37,38]. These findings emphasise the limitations of relying solely on protein expression as a surrogate for underlying genomic events.

Importantly, sequencing-based MSI assessment demonstrated exceptionally high concordance with conventional MMR IHC while also identifying a small subset of MSI-high tumours with retained MMR protein expression [37,38]. This observation suggests that genomic MSI testing may serve as a valuable confirmatory tool in cases showing equivocal, unexpected, or discordant MMR IHC results, particularly when therapeutic decisions regarding immune checkpoint inhibitors depend on accurate MSI classification.

Similarly, genomic profiling provided superior detection of gene fusions, exon-skipping events, and copy-number alterations that may not reliably translate into detectable protein overexpression. In contrast, IHC retained distinct advantages by preserving tissue architecture, allowing assessment of biomarker expression, and providing rapid and cost-effective evaluation within routine diagnostic workflows. Therefore, rather than representing competing technologies, IHC and genomic profiling should be viewed as complementary diagnostic modalities. Their integrated application offers the greatest diagnostic accuracy, improves molecular classification, and enhances patient selection for precision oncology therapies [24,25,29,31,37,38].

Based on the collective findings of the included studies, a practical biomarker-specific testing approach is proposed to guide clinical decision-making and complement, rather than replace established tumour-specific clinical practice guidelines, as summarised in Table 3.

**Table 3:** Suggested biomarker-specific testing approach based on the reviewed evidence Practical biomarker-specific testing recommendations derived from the evidence synthesised in this systematic review. The table summarises suggested first-line diagnostic approaches, indications for complementary genomic profiling, and evidence-based recommendations for integrating immunohistochemistry and genomic profiling according to tumour type and biomarker characteristics. Recommendations are intended to support clinical decision-making and should be interpreted in conjunction with current tumour-specific clinical practice guidelines. IHC, immunohistochemistry; NGS, next-generation sequencing; MSI, microsatellite instability; MMR, mismatch repair; ER, estrogen receptor; PR, progesterone receptor; HRs, hormone receptors; CUP, carcinoma of unknown primary; ISH, in situ hybridization; PCR, polymerase chain reaction; ALK, Anaplastic Lymphoma Kinase; ROS1, ROS Proto-Oncogene 1, Receptor Tyrosine Kinase; HER2, Human Epidermal Growth Factor Receptor 2; PTEN, Phosphatase and Tensin Homolog; EGFR, Epidermal Growth Factor Receptor; BRAF, B-Raf Proto-Oncogene, Serine/Threonine Kinase; PD-L1, programmed cell death ligand 1; NSCLC, Non-Small Cell Lung Cancer.

Biomarker/Tumour	Suggested first-line test	When should genomic profiling be added	Recommendation
HER2 – Breast cancer [13,26,30,32,34]	IHC with reflex FISH for equivocal (2+) cases	Discordant IHC/FISH results, comprehensive genomic profiling, clinical trial eligibility	IHC remains first-line; NGS is complementary in selected cases.
HER2 – Gastric cancer [26,30,32,34]	IHC with confirmatory ISH/FISH	Equivocal or heterogeneous staining, treatment-resistant disease	Combining IHC and genomic testing is recommended because of tumour heterogeneity.
ALK – NSCLC [18,24,25,34]	Validated ALK IHC	Negative/equivocal IHC with strong clinical suspicion, uncommon fusions, comprehensive molecular profiling	NGS is recommended to confirm and detect atypical ALK rearrangements.
ROS1 – NSCLC [18,24,34]	ROS1 IHC screening	Positive IHC requires molecular confirmation	NGS or another molecular method should confirm ROS1 positivity before targeted therapy.
EGFR – NSCLC [18,24,34]	Molecular testing (NGS or PCR)	Routine for advanced disease	Genomic testing is preferred because IHC cannot reliably detect EGFR mutations.
MMR/MSI – Colorectal & Endometrial [5,17,28,31,37,38]	MMR IHC	Discordant/equivocal IHC, suspected Lynch syndrome, immunotherapy decisions	NGS-MSI is recommended as a complementary or confirmatory test.
PTEN [17]	IHC	Abnormal or equivocal IHC, research settings, therapeutic stratification	NGS should complement PTEN IHC because protein loss does not consistently reflect mutation status.
ER / PR – Breast cancer [13,29,35,36]	IHC	Selected research or complex cases	IHC remains the standard; RNA-based assays may provide complementary quantitative assessment.
Ki-67 [29,35,36]	IHC	Selected research or prognostic applications	RNA-based methods may improve reproducibility but are not routine replacements.
PD-L1 [6,14,15,29,35]	Standardised PD-L1 IHC (tumour-specific scoring system)	Discordant results, comprehensive molecular profiling, research	IHC remains the clinical standard; genomic profiling provides complementary predictive information but does not replace PD-L1 testing.
Carcinoma of Unknown Primary (CUP) [33]	Sequential IHC panel	Persistent diagnostic uncertainty	Gene-expression profiling or comprehensive genomic profiling should be considered after inconclusive IHC.

In accordance with the reviewed evidence, the optimal diagnostic strategy depends on the biomarker, tumour type, and clinical context. IHC remains the preferred first-line method for most routinely assessed biomarkers because of its accessibility, rapid turnaround time, and cost-effectiveness. Genomic profiling should be incorporated when IHC findings are equivocal or discordant, when biomarkers lack reliable protein surrogates, or when comprehensive molecular characterisation is required to guide precision oncology.

Discussion

This systematic review demonstrates that the optimal use of IHC and genomic profiling depends on the biomarker, tumour type, and clinical context rather than the superiority of one diagnostic modality over the other. Across the included studies, IHC remained the preferred first-line method for routine biomarker assessment because of its accessibility, rapid turnaround time, and established diagnostic performance. However, genomic profiling consistently provided additional clinically relevant information by detecting actionable molecular alterations, resolving diagnostically challenging cases, and refining molecular classification beyond protein expression alone [24-38].

The degree of diagnostic agreement between IHC and genomic profiling varied according to the biomarker evaluated. Excellent agreement was observed for MMR/MSI testing and hormone receptor assessment in breast cancer, whereas biomarkers such as PTEN, PD-L1, and HER2 in gastric and other non-breast malignancies demonstrated greater biological and technical complexity. These findings emphasise that protein expression does not always accurately reflect the underlying genomic alterations and support a complementary rather than replacement role for genomic profiling in selected clinical scenarios [24-38].

From a clinical perspective, the reviewed evidence supports a context-specific diagnostic strategy in which genomic profiling is incorporated when IHC findings are equivocal or discordant, when biomarkers lack reliable protein surrogates, or when comprehensive molecular characterisation is required for therapeutic decision-making. The practical biomarker-specific recommendations proposed in Table 3 synthesise these findings into an evidence-based framework that complements current tumour-specific clinical practice guidelines rather than replacing them [5,13,17,18,24-38].

Limitations and Future Directions

This review's findings should be interpreted while considering several important limitations. Considerable heterogeneity existed among the included studies regarding tumour types, biomarkers evaluated, genomic platforms employed, study populations, and methodological approaches. Given the heterogeneity of the included studies, meta-analysis was not feasible, and the findings were synthesised narratively. Most included studies were retrospective, introducing an inherent risk of selection bias that should be considered when interpreting the findings. Publication bias was not formally assessed because no meta-analysis was performed, and formal GRADE assessment was not undertaken owing to the substantial methodological heterogeneity among the included studies. Inconsistencies in the reporting of pre-analytical factors, assay methodologies, scoring criteria, sequencing depth, and diagnostic thresholds further complicated cross-study comparisons. Specifically, some studies reported concordance percentages, whereas others presented correlation coefficients, diagnostic accuracy, κ statistics, or predictive values, limiting direct quantitative comparison. Despite these limitations, the available evidence consistently supports the complementary roles of immunohistochemistry and genomic profiling in precision oncology. Future research should focus on well-designed prospective studies with standardised methodologies, tumour-specific analyses, and clinically meaningful endpoints. Additional investigations evaluating cost-effectiveness, accessibility, turnaround time, and real-world clinical outcomes will be essential to optimise evidence-based diagnostic algorithms and facilitate the integration of molecular testing into routine cancer care across diverse healthcare settings.

In addition to the limitations of the included studies, this review has several methodological limitations. The search was restricted to English-language publications, and the review protocol was not prospectively registered, which may have introduced language bias and reduced methodological transparency. Relevant unpublished or grey literature may also not have been captured.

## Conclusions

The reviewed evidence indicates that IHC and genomic profiling fulfil complementary rather than competing roles in modern cancer diagnostics. IHC remains an accessible and efficient first-line technique for tumour classification and biomarker assessment, whereas genomic profiling captures a wider spectrum of molecular abnormalities. The included studies demonstrate that integrating these approaches enhances diagnostic accuracy, improves molecular classification, and expands opportunities for personalised treatment selection. This integrated strategy is particularly valuable in tumours with biologically complex profiles, equivocal IHC findings, or biomarkers known to exhibit significant genotype-phenotype discordance.

As molecularly guided treatment continues to progress, future diagnostic pathways should adopt context-specific testing strategies that combine histomorphology, immunophenotyping, and genomic information. The proposed biomarker-specific recommendations provide a practical framework for integrating IHC and genomic profiling into routine diagnostic practice while complementing established tumour-specific guidelines. Such an approach, supported by multidisciplinary collaboration, evidence-based guidelines, and appropriate laboratory infrastructure, has the potential to optimise patient stratification, reduce diagnostic uncertainty, and improve clinical outcomes across diverse health care settings.

## Appendices

Appendix A: Full database-specific search strategies

Systematic Review Title: Immunohistochemistry (IHC) Versus Genomic Profiling in Cancer: Roles in Precision Medicine

PRISMA 2020 Item 7 Compliance

Search Period: 1 January 2013 to 31 December 2025

Date of Final Search: 31 December 2025

Language Restriction: English

Population Restriction: Human studies

Databases Searched:

1. PubMed (MEDLINE)

2. Scopus

3. Web of Science Core Collection

Database 1: PubMed

Date searched:

31 December 2025

Search field:

Title/Abstract

Search strategy:

((“immunohistochemistry”[Title/Abstract] OR “IHC”[Title/Abstract]) AND (“genomic profiling”[Title/Abstract] OR “molecular profiling”[Title/Abstract] OR “next-generation sequencing”[Title/Abstract] OR “NGS”[Title/Abstract] OR “RNA sequencing”[Title/Abstract]) AND (“cancer”[Title/Abstract] OR “tumour”[Title/Abstract] OR “tumor”[Title/Abstract] OR “malignancy”[Title/Abstract] OR “oncology”[Title/Abstract]))

Filters applied:

AND (“2013/01/01”[Date - Publication] : “2025/12/31”[Date - Publication])

AND Humans[Mesh]

AND English[lang]

Additional targeted PubMed searches:

(“HER2”[Title/Abstract] AND sequencing[Title/Abstract])

(“ALK rearrangement”[Title/Abstract] AND NGS[Title/Abstract])

(“ROS1”[Title/Abstract] AND NGS[Title/Abstract])

(“mismatch repair”[Title/Abstract] AND MSI sequencing[Title/Abstract])

(“precision oncology”[Title/Abstract] AND genomic profiling[Title/Abstract])

Database 2: Scopus

Date searched:

31 December 2025

Search field:

TITLE-ABS-KEY

Search strategy:

TITLE-ABS-KEY

( (immunohistochemistry OR IHC)

AND (genomic profiling

OR molecular profiling

OR next-generation sequencing

OR NGS

OR RNA sequencing)

AND

(cancer

OR tumour

OR tumor

OR malignancy

OR oncology) )

Filters applied:

PUBYEAR > 2012

AND PUBYEAR < 2026

AND LANGUAGE(english)

Additional targeted Scopus searches:

TITLE-ABS-KEY(“HER2” AND sequencing)

TITLE-ABS-KEY(“ALK rearrangement” AND NGS)

TITLE-ABS-KEY(“ROS1” AND NGS)

TITLE-ABS-KEY(“mismatch repair” AND “MSI sequencing”)

TITLE-ABS-KEY(“precision oncology” AND “genomic profiling”)

Database 3: Web of Science Collection

Date searched:

31 December 2025

Search field:

Topic Search (TS)

Search strategy:

TS=

( (immunohistochemistry OR IHC)

AND

(genomic profiling

OR molecular profiling

OR next-generation sequencing

OR NGS

OR RNA sequencing)

AND

(cancer

OR tumour

OR tumor

OR malignancy

OR oncology) )

Filters applied:

Timespan: 2013-2025

Language:

English

Indexes searched: Science Citation Index Expanded (SCI-EXPANDED)

Emerging Sources Citation Index (ESCI)

Additional targeted Web of Science searches:

TS=(“HER2” AND sequencing)

TS=(“ALK rearrangement” AND NGS)

TS=(“ROS1” AND NGS)

TS=(“mismatch repair” AND “MSI sequencing”)

TS=(“precision oncology” AND “genomic profiling”)

STUDY SELECTION WORKFLOW

·         Records identified from database searching: n=423

·         Duplicate records removed: n=106

·         Records screened by title and abstract: n=317

·         Records excluded during screening: n=230

·         Reports sought for retrieval: n=87

·         Reports not retrieved: n=28

·         Full-text reports assessed for eligibility: n=59

·         Full-text reports excluded: n=44

·         Studies included in qualitative synthesis: n=15

ELIGIBILITY CRITERIA

Inclusion Criteria

·         Peer-reviewed studies published between January 2013 and December 2025.

·         Human studies published in English.

·         Studies directly comparing immunohistochemistry (IHC) with at least one genomic profiling approach.

·         Genomic methods including next-generation sequencing (NGS), comprehensive genomic profiling, RNA sequencing, gene-expression profiling, copy-number variation analysis, or sequencing-based microsatellite instability assessment.

·         Studies reporting diagnostic performance, concordance, discordance, or clinical implications of IHC and genomic testing.

Exclusion Criteria

·         Non-comparative studies.

·         Conference abstracts.

·         Narrative reviews.

·         Editorials, commentaries, and letters.

·         Methodological studies without clinical comparison.

·         Studies evaluating only immunohistochemistry.

·         Studies evaluating only genomic testing.

·         Non-English publications.

·         animal or in vitro studies.

Appendix B: Review process

All records retrieved from the databases were imported into a reference management system. Duplicate records were removed before screening. Title and abstract screening was performed manually by the review authors. Potentially eligible studies underwent full-text review according to predefined eligibility criteria. No automation tools, artificial intelligence-assisted screening systems, or machine-learning selection methods were used during study identification, screening, eligibility assessment, or study inclusion.

The review was conducted and reported in accordance with the Preferred Reporting Items for Systematic Reviews (PRISMA) 2020 Statement.

**Table 4:** Detailed CASP-based methodological quality assessment of the included studies (n=15) Methodological quality and risk-of-bias assessment of the included studies (n=15) based on the Critical Appraisal Skills Programme (CASP) Diagnostic Test Accuracy Checklist. Assessments were performed independently by two reviewers using a qualitative appraisal approach evaluating study objectives, appropriateness of study design, participant selection, validity of immunohistochemical and genomic methodologies, outcome reporting, applicability, and potential sources of bias. Disagreements were resolved through discussion and consensus. CASP was applied as a qualitative appraisal framework rather than a quantitative scoring instrument. Risk-of-bias and overall-quality categories were assigned through consensus evaluation of methodological strengths and limitations across the assessed CASP domains. No automation tools or artificial intelligence-assisted methods were used during the quality assessment process. CASP, Critical Appraisal Skills Programme; IHC, Immunohistochemistry; NGS, Next-Generation Sequencing; MSI, Microsatellite Instability; MMR, Mismatch Repair.

Study	Design	Focused Question	Appropriate Design	Participant Selection	Valid IHC	Valid Genomic Method	Outcome Reporting	Applicability	Risk of Bias	Overall Quality	Comments/Justification
Nong et al. 2019 [24]	Retrospective comparative	Yes	Yes	Yes	Yes	Yes	Yes	High	Low	High	Clear objectives; validated IHC and targeted NGS; clinically relevant NSCLC biomarkers; moderate sample size.
Zeng et al. 2020 [25]	Retrospective cohort	Yes	Yes	Yes	Yes	Yes	Yes	High	Low	High	Dual-testing design with treatment outcomes; validated ALK assays; low methodological concerns.
Niu et al. 2020 [26]	Diagnostic accuracy study	Yes	Yes	Yes	Yes	Yes	Yes	High	Moderate	High	Strong HER2 validation; gastric subgroup relatively small; heterogeneity may affect estimates.
Kim et al. 2023 [27]	Retrospective molecular correlation	Yes	Yes	Moderate	Yes	Yes	Yes	Moderate	Moderate	Moderate	Small sample size (n=18); exploratory PTEN analysis; findings clinically useful but limited power.
Xiao et al. 2021 [28]	Comparative diagnostic study	Yes	Yes	Yes	Yes	Yes	Yes	High	Low	High	Large cohort with MSI/MMR validation against PCR and NGS; robust methodology.
Sorokin et al. 2020 [29]	Comparative biomarker study	Yes	Yes	Moderate	Yes	Yes	Yes	High	Moderate	High	Small cohorts but strong RNA-seq/IHC correlations; limited generalizability.
Kıvrak et al. 2022 [30]	Retrospective validation study	Yes	Yes	Moderate	Yes	Yes	Yes	Moderate	Moderate	Moderate	Limited amplified cases; HER2 assessment strengthened by SISH confirmation.
Rios-Doria et al. 2023 [31]	Comparative molecular classification	Yes	Yes	Yes	Yes	Yes	Yes	High	Low	High	Large endometrial cancer cohort; integrated classification approach; low risk of bias.
Shah et al. 2022 [32]	Multi-tumour validation study	Yes	Yes	Yes	Yes	Yes	Yes	High	Moderate	High	Strong clinical applicability; variability across tumour types introduces some heterogeneity.
Handorf et al. 2013 [33]	Multicentre comparative study	Yes	Yes	Yes	Yes	Yes	Yes	High	Moderate	High	Multicentre design improves validity; older technology limits current applicability.
Choi et al. 2022 [34]	Comparative genomic validation	Yes	Yes	Yes	Yes	Yes	Yes	High	Low	High	Large multi-cancer cohort; orthogonal validation methods support findings.
Kushnarev et al. 2025 [35]	Comparative biomarker study	Yes	Yes	Yes	Yes	Yes	Yes	High	Moderate	High	Strong biomarker correlations; tumour purity may influence RNA-seq results.
Sinn et al. 2017 [36]	Comparative diagnostic study	Yes	Yes	Moderate	Yes	Yes	Yes	Moderate	Moderate	Moderate	Limited sample details in abstract; focused breast cancer biomarker validation.
Ali-Fehmi et al. 2024 [37]	Large-scale concordance study	Yes	Yes	Yes	Yes	Yes	Yes	High	Low	High	Very large cohort (>190,000 cases); excellent concordance assessment.
Aydın Meriçöz et al. 2025 [38]	Comparative MSI study	Yes	Yes	Moderate	Yes	Yes	Yes	High	Moderate	High	Moderate sample size; valuable confirmation of MSI/MMR discordance.

Appendix C 

**Table 5:** Full-text studies excluded after eligibility assessment Excluded References (1-44) (Excluded after full-text analysis)

No.	Reference (complete detail)	Reason for exclusion
1	K. Brisudova et al., "Gene rearrangement detection by next-generation sequencing in patients with non-small cell lung carcinoma," Biomedical Papers, 2020. doi: 10.5507/BP.2020.015.	Narrative review without direct clinical comparison between IHC and genomic testing.
2	M. S. Tsao et al., "Old soldiers never die: is there still a role for immunohistochemistry in the era of next-generation sequencing panel testing," Journal of Thoracic Oncology, 2019. doi: 10.1016/J.JTHO.2019.09.007.	Narrative review without original comparative clinical data.
3	Kim CH, Kim SH, Park SY, Yoo J, Kim SK, Kim HK. Identification of EGFR Mutations by Immunohistochemistry with EGFR Mutation-Specific Antibodies in Biopsy and Resection Specimens from Pulmonary Adenocarcinoma. Cancer Res Treat. 2015 Oct;47(4):653-60. doi: 10.4143/crt.2014.118. Epub 2015 Jan 30. PMID: 25687872; PMCID: PMC4614184.	Single diagnostic modality (immunohistochemistry only); no direct comparison with genomic testing.
4	Albuquerque DAN, Vianna MT, Sampaio LAF, Vasiliu A, Neves Filho EHC. Diagnostic accuracy of artificial intelligence in classifying HER2 status in breast cancer immunohistochemistry slides and implications for HER2-low cases: a systematic review and meta-analysis. medRxiv. Published November 4, 2024. doi:10.1101/2024.11.04.24316688	This AI study compares: AI-assisted digital pathology and Human pathologist interpretation ; Both are based on the same IHC slides
5	Torlakovic E, Lim HJ, Adam J, et al. “Interchangeability” of PD-L1 immunohistochemistry assays: a meta-analysis of diagnostic accuracy. Modern Pathology 2020;33:4–17. DOI: 10.1038/s41379-019-0327-4	Narrative review and meta-analysis focused exclusively on IHC assay comparison without genomic testing comparison.
6	T. J. A. Dekker et al., "Determining sensitivity and specificity of HER2 testing in breast cancer using a tissue micro-array approach," Breast Cancer Research, 2012. doi: 10.1186/BCR3208.	Single diagnostic modality (immunohistochemistry only); assay reproducibility study without genomic comparison.
7	Wu S, Yue M, Zhang J, Li X, Li Z, Zhang H, Wang X, Han X, Cai L, Shang J, Jia Z, Wang X, Li J, Liu Y. The role of artificial intelligence in accurate interpretation of HER2 immunohistochemical scores 0 and 1+ in breast cancer. Modern Pathology. 2023;36(3):100054. doi:10.1016/j.modpat.2022.100054.	Evaluated only HER2 IHC interpretation. No genomic testing comparison.
8	M Shibu D. Mutation specific antibodies for immunohistochemical detection of epidermal growth factor receptor mutations in lung adenocarcinomas. J Med Sci Clin Res. 2022;10(2). doi:10.18535/jmscr/v10i2.15.	This study evaluated: Mutation-specific IHC only, no genomic profiling method was performed. .
9	C. Charoenlap et al., "Diagnostic Accuracy and Reproducibility of ChatGPT-4o for HER2 Immunohistochemistry Scoring in Equivocal Breast Cancer Cases," Asian Pacific Journal of Cancer Prevention, vol. 27, no. 5, p. 1703, 2026. doi: 10.31557/APJCP.2026.27.5.1703.	Single diagnostic modality (immunohistochemistry only); AI-based IHC scoring study without genomic comparison.
10	A. E. Canda et al., "Immunohistochemical HER2 Status Evaluation in Breast Cancer Pathology Samples: A Multicenter, Parallel-Design Concordance Study," The Journal of Breast Health, 2018. doi: 10.5152/EJBH.2018.3961.	Single diagnostic modality (immunohistochemistry only); inter-laboratory concordance study without genomic comparison.
11	"Multi-institutional Assessment of Pathologist scoring HER2 Immunohistochemistry," Research Square preprint, 2022. doi: 10.21203/rs.3.rs-1763087/v1.	Single diagnostic modality (immunohistochemistry only); preprint without genomic comparison.
12	Che J, Zhang X, et al. Validation of a targeted sequencing panel with automatic analysis system for clinical decision support in cancer therapy. Research Square. Preprint. Published October 28, 2023. doi:10.21203/rs.3.rs-3482627/v1	Evaluates a genomic profiling platform (NGS), Reports molecular findings and panel validation, Does not compare NGS results with immunohistochemistry (IHC), validation study without IHC comparison.
13	V. Pestinger et al., "Use of an Integrated Pan-Cancer Oncology Enrichment Next-Generation Sequencing Assay to Measure Tumour Mutational Burden and Detect Clinically Actionable Variants," Molecular Diagnosis & Therapy, 2020. doi: 10.1007/S40291-020-00462-X.	Single diagnostic modality (genomic testing only); NGS assay-focused study without IHC comparison.
14	T. Akahane et al., "Endometrial Liquid-Based Cytology Specimens Preserve High Genome Quality for Molecular Classification After Long-Term Storage," Diagnostic Cytopathology, 2025. doi: 10.1002/dc.70008.	Single diagnostic modality (genomic testing only); specimen quality control study without IHC comparison.
15	T. Eifert et al., "Clinical application of a cancer genomic profiling assay to guide precision medicine decisions," Personalized Medicine, 2017. doi: 10.2217/PME-2017-0011.	Single diagnostic modality (genomic testing only); clinical application study without IHC comparison.
16	Christgen M, van Luttikhuizen JL, Raap M, et al. Precise ERBB2 copy number assessment in breast cancer by means of molecular inversion probe array analysis. Oncotarget. 2016;7:82733-82740. doi:10.18632/oncotarget.12421.	Methodological diagnostic comparison without direct IHC–genomic comparison.
17	J. S. Saldivar et al., "Analytic validation of NeXT Dx™, a comprehensive genomic profiling assay," Oncotarget, 2023. doi: 10.18632/oncotarget.28490.	Analytical validation study evaluating a genomic profiling platform only; no immunohistochemistry comparator and no direct IHC-versus-genomic comparison.
18	Choi YW, Kim JH, Park JH, et al. Tumor Heterogeneity Index to Detect Human Epidermal Growth Factor Receptor 2 Amplification by Next-Generation Sequencing: A Direct Comparison Study with Immunohistochemistry. *J Mol Diagn*. 2019. doi: 10.1016/j.jmoldx.2019.02.007	A single-biomarker methodological validation study and lacking broader clinical comparison outcomes.
19	D. Fabrizio et al., "Beyond microsatellite testing: assessment of tumor mutational burden identifies subsets of colorectal cancer who may respond to immune checkpoint inhibition," Journal of Gastrointestinal Oncology, 2018. doi: 10.21037/JGO.2018.05.06.	Single diagnostic modality (genomic testing only); genomic biomarker study without IHC comparison.
20	S. J. Klempner et al., "PD-L1 Immunohistochemistry in Gastric Cancer: Comparison of Combined Positive Score and Tumor Area Positivity Across 28-8, 22C3, and SP263 Assays," JCO Precision Oncology, 2024. doi: 10.1200/po.24.00230.	Single diagnostic modality (immunohistochemistry only); IHC assay comparison study without genomic testing comparison.
21	Y. Zhao et al., "Concordance of PD-L1 Status Between Image-Guided Percutaneous Biopsies and Matched Surgical Specimen in Non-Small Cell Lung Cancer," Frontiers in Oncology, vol. 10, article 551367, 2021. doi: 10.3389/FONC.2020.551367.	Single diagnostic modality (immunohistochemistry only); biopsy-surgical specimen concordance study without genomic comparison.
22	Solomon BJ, Mok T, Kim DW, et al. First-line crizotinib versus chemotherapy in ALK-positive lung cancer. *N Engl J Med*. 2014;371(23):2167-2177. doi: 10.1056/NEJMoa1408440	This study does not directly compare Immunohistochemistry (IHC) with genomic profiling or NGS. Its primary objective was: Treatment efficacy comparison and Crizotinib versus chemotherapy
23	A. Sinclair et al., "Interobserver agreement in programmed cell death-ligand 1 immunohistochemistry scoring in nonsmall cell lung carcinoma cytologic specimens," Diagnostic Cytopathology, 2021. doi: 10.1002/DC.24651.	Single diagnostic modality (immunohistochemistry only); inter-observer agreement study without genomic comparison.
24	P. Yuan et al., "The Reproducibility of Histopathologic Assessments of Programmed Cell Death-Ligand 1 Using Companion Diagnostics in NSCLC," JTO Clinical and Research Reports, vol. 2, article 100102, 2021. doi: 10.1016/J.JTOCRR.2020.100102.	Single diagnostic modality (immunohistochemistry only); reproducibility study of IHC companion diagnostics without genomic comparison.
25	B. Schoemig-Markiefka et al., "Optimized PD-L1 scoring of gastric cancer," Gastric Cancer, 2021. doi: 10.1007/S10120-021-01195-4.	Single diagnostic modality (immunohistochemistry only); IHC scoring optimization study without genomic comparison.
26	S. Ahn et al., "Best Practice PD-L1 Staining and Interpretation in Gastric Cancer Using PD-L1 IHC PharmDx 22C3 and PD-L1 IHC PharmDx 28-8 Assays, with Reference to Common Issues and Solutions," Biomedicines, 2025. doi: 10.3390/biomedicines13112824.	Narrative review focused on IHC best practices without original comparative clinical data.
27	M. Zaakouk et al., "Inter- and Intra-Observer Agreement of PD-L1 SP142 Scoring in Breast Carcinoma-A Large Multi-Institutional International Study," Cancers, vol. 15, article 1511, 2023. doi: 10.3390/cancers15051511.	Single diagnostic modality (immunohistochemistry only); inter-observer agreement study without genomic comparison.
28	E. Hacihasanoglu et al., "PD-L1 Assessment in Needle Core Biopsies of Non-Small Cell Lung Cancer: Interpathologist Agreement and Potential Associated Histopathological Features," Turkish Journal of Pathology, 2024. doi: 10.5146/tjpath.2023.01609.	Single diagnostic modality (immunohistochemistry only); inter-pathologist agreement study without genomic comparison.
29	D. L. Rimm et al., "A prospective, multi-institutional, pathologist-based assessment of 4 immunohistochemistry assays for PD-L1 expression in non-small cell lung cancer," JAMA Oncology, 2017. doi: 10.1001/JAMAONCOL.2017.0013.	Single diagnostic modality (immunohistochemistry only); multi-institutional IHC assay assessment without genomic comparison.
30	M. Zhang et al., "High concordance of programmed death-ligand 1 expression with immunohistochemistry detection between antibody clones 22C3 and E1L3N in non-small cell lung cancer biopsy samples," Translational Cancer Research, 2020. doi: 10.21037/TCR-20-101.	Compared two IHC antibody clones only; no genomic profiling method evaluated
31	S. K. Lou et al., "Implementation of PD-L1 22C3 IHC pharmDxTM in Cell Block Preparations of Lung Cancer: Concordance with Surgical Resections and Technical Validation of CytoLyt® Prefixation," Acta Cytologica, 2020. doi: 10.1159/000508628.	Single diagnostic modality (immunohistochemistry only); technical validation study without genomic comparison.
32	S. Ahn et al., "Programmed Death Ligand 1 Immunohistochemistry in Triple-Negative Breast Cancer: Evaluation of Inter-Pathologist Concordance and Inter-Assay Variability," Journal of Breast Cancer, vol. 24, article E29, 2021. doi: 10.4048/JBC.2021.24.E29.	Single diagnostic modality (immunohistochemistry only); inter-pathologist concordance study without genomic comparison.
33	W. Li et al., "NGS-based oncogenic mutations analysis in advanced colorectal cancer patients improves targeted therapy prediction," Pathology Research and Practice, 2019. doi: 10.1016/J.PRP.2018.12.037.	Genomic profiling study only; no direct comparison with immunohistochemistry; did not evaluate concordance or diagnostic performance between IHC and genomic testing
34	W. Hou et al., "Targeted next generation sequencing in Chinese colorectal cancer patients guided anti-EGFR treatment and facilitated precision cancer medicine," Oncotarget, 2017. doi: 10.18632/ONCOTARGET.21349.	Single diagnostic modality (genomic testing only); targeted NGS study without IHC comparison.
35	N. Knijn et al., "Sequencing of RAS/RAF pathway genes in primary colorectal cancer and matched liver and lung metastases," Applied Cancer Research, 2019. doi: 10.1186/S41241-019-0079-Y.	Single diagnostic modality (genomic testing only); RAS/RAF pathway sequencing study without IHC comparison.
36	W. Li et al., "Prevalence and characteristics of PIK3CA mutation in mismatch repair-deficient colorectal cancer," Journal of Cancer, 2020. doi: 10.7150/JCA.37437.	Single diagnostic modality (genomic testing only); PIK3CA mutation prevalence study without IHC comparison.
37	Y. J. Choi et al., "Genomic Profiling of Blood-Derived Circulating Tumor DNA from Patients with Colorectal Cancer: Implications for Response and Resistance to Targeted Therapeutics," Molecular Cancer Therapeutics, 2019. doi: 10.1158/1535-7163.MCT-18-0965.	Single diagnostic modality (genomic testing only); circulating tumor DNA profiling study without IHC comparison.
38	Z. Liu et al., "Combined Detection of KRAS, NRAS, BRAF and PIK3CA Mutations in the Plasma and Tumor Tissues of Colorectal Cancer Patients," Chinese Medical Journal, 2019. doi: 10.3760/cma.j.issn.0529-5807.2019.05.008.	Single diagnostic modality (genomic testing only); multi-gene mutation detection study without IHC comparison.
39	F. Guo et al., "Identification of molecular subtypes for endometrial carcinoma using a 46-gene next-generation sequencing panel: a retrospective study on a consecutive cohort," ESMO Open, vol. 9, article 103710, 2024. doi: 10.1016/j.esmoop.2024.103710.	Integrated molecular classification study rather than a direct comparative diagnostic study.
40	F. Torricelli et al., "Computational development of a molecular-based approach to improve risk stratification of endometrial cancer patients," Oncotarget, 2018. doi: 10.18632/ONCOTARGET.25354.	Evaluated genomic profiling (NGS) for prognostic stratification without direct comparison to immunohistochemistry; did not assess IHC–genomic concordance or diagnostic performance
41	L. Reggiani Bonetti et al., "Clinical Impact and Prognostic Role of KRAS/BRAF/PIK3CA Mutations in Stage I Colorectal Cancer," Disease Markers, vol. 2018, article 2959801, 2018. doi: 10.1155/2018/2959801.	Single diagnostic modality (genomic testing only); prognostic mutation study without IHC comparison.
42	Y. Lee et al., "Clinical Application of Targeted Deep Sequencing in Metastatic Colorectal Cancer Patients: Actionable Genomic Alteration in K-MASTER Project," Cancer Research and Treatment, 2021. doi: 10.4143/CRT.2020.559.	Single diagnostic modality (genomic testing only); targeted deep sequencing study without IHC comparison.
43	A. Bayle et al., "Next-Generation Sequencing Targeted Panel in Routine Care for Metastatic Colon Cancers," Cancers, vol. 13, article 5750, 2021. doi: 10.3390/cancers13225750.	Single diagnostic modality (genomic testing only); NGS panel implementation study without IHC comparison.
44	G. Bruera et al., "KRAS, NRAS and BRAF mutations detected by next generation sequencing, and differential clinical outcome in metastatic colorectal cancer patients treated with first line FIr-B/FOx adding bevacizumab to triplet chemotherapy," Oncotarget, 2018. doi: 10.18632/ONCOTARGET.25180.	Single diagnostic modality (genomic testing only); NGS mutation detection and clinical outcome study without IHC comparison.
